# Socioeconomic status and dietary patterns in children from around the world: different associations by levels of country human development?

**DOI:** 10.1186/s12889-017-4383-8

**Published:** 2017-05-16

**Authors:** Taru Manyanga, Mark S. Tremblay, Jean-Philippe Chaput, Peter T. Katzmarzyk, Mikael Fogelholm, Gang Hu, Rebecca Kuriyan, Anura Kurpad, Estelle V. Lambert, Carol Maher, Jose Maia, Victor Matsudo, Timothy Olds, Vincent Onywera, Olga L. Sarmiento, Martyn Standage, Catrine Tudor-Locke, Pei Zhao, Vera Mikkila, Stephanie T. Broyles, for the ISCOLE Research Group, Peter T. Katzmarzyk, Timothy S. Church, Denise G. Lambert, Tiago Barreira, Stephanie Broyles, Ben Butitta, Catherine Champagne, Shannon Cocreham, Kara D. Denstel, Katy Drazba, Deirdre Harrington, William Johnson, Dione Milauskas, Emily Mire, Allison Tohme, Ruben Rodarte, Bobby Amoroso, John Luopa, Rebecca Neiberg, Scott Rushing, Timothy Olds, Carol Maher, Lucy Lewis, Katia Ferrar, Effie Georgiadis, Rebecca Stanley, Victor Keihan Rodrigues Matsudo, Sandra Matsudo, Timoteo Araujo, Luis Carlos de Oliveira, Luis Fabiano, Diogo Bezerra, Gerson Ferrari, Mark S. Tremblay, Jean-Philippe Chaput, Priscilla Bélanger, Mike Borghese, Charles Boyer, Allana LeBlanc, Claire Francis, Geneviève Leduc, Pei Zhao, Gang Hu, Chengming Diao, Wei Li, Weiqin Li, Enqing Liu, Gongshu Liu, Hongyan Liu, Jian Ma, Yijuan Qiao, Huiguang Tian, Yue Wang, Tao Zhang, Fuxia Zhang, Olga Sarmiento, Julio Acosta, Yalta Alvira, Maria Paula Diaz, Rocio Gamez, Maria Paula Garcia, Luis Guillermo Gómez, Lisseth Gonzalez, Silvia Gonzalez, Carlos Grijalba, Leidys Gutierrez, David Leal, Nicolas Lemus, Etelvina Mahecha, Maria Paula Mahecha, Rosalba Mahecha, Andrea Ramirez, Paola Rios, Andres Suarez, Camilo Triana, Mikael Fogelholm, Elli Hovi, Jemina Kivelä, Sari Räsänen, Sanna Roito, Taru Saloheimo, Leena Valta, Anura Kurpad, Rebecca Kuriyan, Deepa P. Lokesh, Michelle Stephanie D’Almeida, Mattilda R. Annie, Lygia Correa, Vijay Dakshina Murthy, Vincent Onywera, Lucy-Joy Wachira, Stella Muthuri, Jose Maia, Alessandra da Silva Borges, Sofia Oliveira Sá Cachada, Raquel Nichele de Chaves, Thayse Natacha Queiroz Ferreira Gomes, Sara Isabel Sampaio Pereira, Daniel Monteiro de Vilhena e Santos, Fernanda Karina dos Santos, Pedro Gil Rodrigues da Silva, Michele Caroline de Souza, Vicki Lambert, Matthew April, Monika Uys, Nirmala Naidoo, Nandi Synyanya, Madelaine Carstens, Martyn Standage, Sean Cumming, Clemens Drenowatz, Lydia Emm, Fiona Gillison, Julia Zakrzewski, Catrine Tudor-Locke, Ashley Braud, Sheletta Donatto, Corbin Lemon, Ana Jackson, Ashunti Pearson, Gina Pennington, Daniel Ragus, Ryan Roubion, John Schuna, Derek Wiltz, Alan Batterham, Jacqueline Kerr, Michael Pratt, Angelo Pietrobelli

**Affiliations:** 1 Children’s Hospital of Eastern Ontario Research Institute, 401 Smyth Road, Ottawa, K1H 8L1 Canada; 2 Pennington Biomedical Research Center, Baton Rouge, USA; 3 University of Helsinki, Helsinki, Finland; 4 St. Johns Research Institute, Bangalore, India; 5 University of Cape Town, Cape Town, South Africa; 6 University of South Australia, Adelaide, Australia; 7 CIFI2D, University of Porto, Porto, Portugal; 8 Center of Studies from the Physical Fitness Research Laboratory, de São Caetano do Sul, Sao Paulo, Brazil; 9 Kenyatta University, Nairobi, Kenya; 10 Universidad de los Andes, Bogota, Colombia; 11 University of Bath, Bath, UK; 12 University of Massachusetts Amherst, Amherst, USA; 13 Tianjin Women’s and Children’s Health Center, Tianjin, China; 14 Academy of Finland, Health Research Unit, Helsinki, Finland

**Keywords:** Unhealthy/healthy diet, Household income, Hdi, Gini index, Non-communicable diseases

## Abstract

**Background:**

Although ‘unhealthy’ diet is a well-known risk factor for non-communicable diseases, its relationship with socio-economic status (SES) has not been fully investigated. Moreover, the available research has largely been conducted in countries at high levels of human development. This is the first study to examine relationships among dietary patterns and SES of children from countries spanning a wide range of human development.

**Methods:**

This was a multinational cross-sectional study among 9–11 year-old children (*n* = 6808) from urban/peri-urban sites across 12 countries. Self-reported food frequency questionnaires were used to determine the children’s dietary patterns. Principal Components Analysis was employed to create two component scores representing ‘unhealthy’ and ‘healthy’ dietary patterns. Multilevel models accounting for clustering at the school and site level were used to examine the relationships among dietary patterns and SES.

**Results:**

The mean age of participants in this study (53.7% girls) was 10.4 years. Largest proportions of total variance in dietary patterns occurred at the individual, site, and school levels (individual, school, site: 62.8%; 10.8%; 26.4% for unhealthy diet pattern (UDP) and 88.9%; 3.7%; 7.4%) for healthy diet pattern (HDP) respectively. There were significant negative ‘unhealthy’ diet-SES gradients in 7 countries and positive ‘healthy’ diet-SES gradients in 5. Within country diet-SES gradients did not significantly differ by HDI. Compared to participants in the highest SES groups, unhealthy diet pattern scores were significantly higher among those in the lowest within-country SES groups in 8 countries: odds ratios for Australia (2.69; 95% CI: 1.33–5.42), Canada (4.09; 95% CI: 2.02–8.27), Finland (2.82; 95% CI: 1.27–6.22), USA (4.31; 95% CI: 2.20–8.45), Portugal (2.09; 95% CI: 1.06–4.11), South Africa (2.77; 95% CI: 1.22–6.28), India (1.88; 95% CI: 1.12–3.15) and Kenya (3.35; 95% CI: 1.91–5.87).

**Conclusions:**

This study provides evidence of diet-SES gradients across all levels of human development and that lower within-country SES is strongly related to unhealthy dietary patterns. Consistency in within-country diet-SES gradients suggest that interventions and public health strategies aimed at improving dietary patterns among children may be similarly employed globally. However, future studies should seek to replicate these findings in more representative samples extended to more rural representation.

**Electronic supplementary material:**

The online version of this article (doi:10.1186/s12889-017-4383-8) contains supplementary material, which is available to authorized users.

## Background

The role of diet in the prevention of non-communicable diseases (NCDs) is well documented [1–5]. Studies in high income countries attribute disparities in obesity and health in part to differences in diet quality [6–8]. For most high income countries in general, energy-dense foods cost less, whereas healthier foods tend to cost more [7, 9, 10]; thus, diet quality may differ by socioeconomic status (SES) [9, 11]. Some [10–12] but not all [13] studies in high income countries, report that healthier diets are associated with higher levels of SES while unhealthy diets are associated with lower SES.

Consistent with the theory of an epidemiologic transition [14], it is plausible to speculate that a reverse relationship would be observed in low-middle-income countries (LMICs), where rapid urbanization has accelerated a nutrition transition [2, 15]. In this setting, high income groups may consume a more energy-dense or unhealthy diet than low-income groups [12, 16]. Our group recently demonstrated the presence of an epidemiological transition in obesity by showing a strong relationship between childhood obesity and SES which was differentially affected by each country’s level of human development (HDI) [17].

Understanding how broader dietary patterns [18] may be associated with SES across countries at different levels of human development, in the context of the ongoing nutrition transition [2, 15] is important to inform public health policies and intervention strategies aimed at preventing NCDs. Level of human development is determined using the HDI, (a summary measure) of average achievement in the three dimensions and calculated as a composite of life expectancy at birth, education and per-capita income [19]. In the present study, dietary pattern is defined as a combination of foods and drinks and their frequency of consumption by the study participants [18].

To the best of our knowledge, no previous study has examined the relationship between SES and dietary patterns in children across multiple countries representing different levels of human development. Moreover, the fact that most of the available evidence has examined these relationships either only in high-income or low-income countries separately, limits our understanding of any similarities or differences across different HDI levels. Therefore, the purpose of the present study was to examine these relationships using reported household income and highest level of parental education as proxies for SES among children from all inhabited continents of the world and explore if the relationships differ across levels of human development. We hypothesized that there would be a gradient in dietary patterns across HDI levels in relation to within-country indicators of SES. We further hypothesized that at higher levels of development, higher within-country SES would be associated with healthy dietary patterns whereas lower SES would be associated with unhealthy dietary patterns; the reverse was hypothesized for countries at lower levels of human development.

## Methods

### Study design and setting

The International Study of Childhood Obesity, Lifestyle and the Environment (ISCOLE) is a cross-sectional, multinational study designed to examine relationships between lifestyle behaviours and obesity among children in urban/peri-urban areas from 256 schools in 12 countries (Australia, Brazil, Canada, China, Colombia, Finland, India, Kenya, Portugal, South Africa, the United Kingdom and the United States of America). These countries differ in several SES indicators across a continuum of HDI and Gini index [20]. Detailed descriptions of the design and methods for ISCOLE have been reported elsewhere [20]. Briefly, the primary sampling frame was schools stratified by an indicator of SES to maximize variability among the recruited schools [20]. A standard protocol was used to collect data across all sites, and all study personnel underwent rigorous training and certification before and continuously during data collection to ensure data quality. Data collection occurred between September 2011 and December 2013. At each study site, data were collected during the school year. In preparing this manuscript, we adhered to the STROBE guidelines/methodology for observational studies.

### Participants

ISCOLE targeted grade levels likely to ensure minimal variability around a mean age of 10 years. All children within the targeted grade level in a sampled school were eligible to participate; hence, the sample included 9–11 year-old children. Based on a priori sample-size and power calculations [20], each site aimed to recruit a gender-balanced sample of at least 500 children. Of the 7372 children who participated in ISCOLE, a total of 6808 remained in the present analytic dataset after excluding participants without valid diet pattern scores (*n* = 172), reported level of parental education (*n* = 361) and body mass index (BMI) z-score (*n* = 31). Except for significantly higher BMI z-scores (*p* = 0.02), descriptive characteristics of participants who were excluded for missing data did not significantly differ from those who were included in the present analysis.

### Measurements

#### Dietary patterns

The current study uses, as dependent variables, scores from two standardized principal components representing a healthy dietary pattern and an unhealthy dietary pattern respectively. Details for deriving these principal components, the scores and all factor loadings for each pattern are provided elsewhere [18]. In brief, a food frequency questionnaire (FFQ) (Additional file 1: Appendix S2) adapted from the Health Behavior in School-aged Children Survey [21] was used to examine ISCOLE participants’ ‘usual’ consumption of 23 different food groups. The FFQ had 7 response categories as follows: never, less than once per week, once per week, 2–4 days per week, 5–6 days per week, once a day every day and more than once a day. Reliability (*r* = 0.52–0.82) of a 15-item version of this FFQ has previously been demonstrated for ranking the frequency of consumption of food items in children [22].

To identify dietary patterns (a combination of foods and drinks and their frequency of consumption by the study participants) among the study population, a principal components analysis (PCA) was carried out using weekly food consumption frequencies as input variables. The PCA was performed first using the total data set and then for each country separately [18]; the dependent variables used in the present study are the component scores derived from the total dataset in order to preserve between-site variability in the measures. The two components were selected for analysis based on eigenvalues and a scree plot analysis [18]. These two components represented an ‘unhealthy diet pattern’ (UDP, with positive loadings for fast food, hamburgers, soft drinks, sweets, fried food etc.) and a ‘healthy diet pattern’ (HDP, with positive loadings for vegetables, fruit, whole grains, low-fat milk etc.) [18]. Diet pattern scores were standardized to ensure normality and higher values for each score represented either an “unhealthier” or “healthier” eating pattern, respectively. Most of the strongly loaded food items in both components were common for all 12 countries [18]. Test-retest reliability of the diet pattern scores developed for ISCOLE indicated moderate-to-strong reliability (ICC = 0.56–0.78) [18].

#### Socioeconomic status

Two different measures of SES were used in the current study: combined annual household income and self-reported highest level of parental education. For the former, participants’ legal guardians/parents self-reported household income using a monetary scale in the currency of each country. Each country-specific income scale (8–10 categories) was collapsed into four levels to facilitate multi-country comparisons [23]. Although not corresponding exactly to quartiles, the four levels were created to ensure the most balanced distribution possible within each country [23]. The development of categories of each country’s income levels has been reported in greater detail by Broyles and colleagues [17]. For education, participants’ legal guardians/parents self-reported (both the mother’s and father’s) highest education levels, which were combined into a single measure indicating highest level of parental education and categorized as ‘did not complete high school’, ‘completed high school or some college’, and ‘bachelor’s or postgraduate degree,’ to be consistent with other ISCOLE studies [24].

#### Country HDI and Gini index

The relationship between dietary patterns and SES across levels of human development (defined above) was examined using the 2011 HDI [25]. To further explore contextual information, the within-country relationships between dietary patterns and SES were assessed across levels of inequality using the Gini index, which reflects the extent to which the distribution of income or consumption expenditure among individuals or households within an economy (i.e., within-country) deviates from a perfectly equal distribution [26]. A Gini index of 0 represents perfect equality whereas an index of 100 represents perfect inequality.

### Covariates

Age, sex, and BMI z-scores were included as covariates in analytic models. Age was computed from birth and observation dates and sex was recorded on a questionnaire. Height and body weight were objectively measured using standard procedures and instrumentation by trained and certified study personnel [20]. Standing height measured without shoes and with participant’s head in the Frankfurt Plane was obtained using a Seca 213 portable stadiometer (Seca Corporation, Hamburg, Germany). After removal of pocket items, shoes and outer clothing, body weight was measured using a Tanita SC-240 bioelectrical impedance analyzer (Tanita Corporation, Tokyo, Japan). Each measurement was repeated and the average of the two was used for analysis. A third measurement was obtained if the first two measurements differed by greater than 0.5 cm or 0.5 kg for height and body mass, respectively. The average of the closest two measurements was used in the analyses. BMI (kg/m^2^) was calculated and age- and sex-specific BMI z-scores were computed using reference data from the World Health Organization [27]. Of note, biological maturity was estimated using the maturity offset method in ISCOLE; however, because age and weight were included in the maturity offset calculation, biological maturity was not included as a covariate in our analyses.

### Treatment of missing income level data

Of the 6808 participants retained in the analytic dataset, 417 (6.1%) were missing data on annual household income. Three sites were missing data in excess of 10%: Brazil (13.0%), Portugal (13.0%), and South Africa (19.4%). Children with missing income data were similar to those with complete data with respect to both outcomes (UDP, *p* = 0.89; HDP, *p* = 0.65). Missing values for income were multiply-imputed (5 imputations) using fully conditional specification (FCS) methods, under missing at random (MAR) assumptions [28] and using SAS version 9.3 (PROC MI). Country-specific models were used to impute income categories according to country-specific income scales. These results were subsequently collapsed into four income levels to facilitate comparisons across countries (described above). All analyses involving income were conducted within each of the five imputed datasets, and results were averaged, and standard errors adjusted appropriately, using the MIANALYZE procedure in SAS. As a sensitivity analysis, we compared results to those resulting from a complete-case analysis. Because results were similar, we have chosen to present those that include the imputed data to maintain a consistent sample size across analyses with either SES measure.

### Statistical analysis

Statistical analyses were performed using SAS version 9.4 (SAS Institute, Cary, NC, USA). Descriptive characteristics (means ± SD) of participants were computed by sex for each study site. Multilevel mixed-effects models (SAS PROC MIXED and PROC GLIMMIX) accounting for clustering at the school and study site levels were used to examine within-country SES gradients in dietary patterns. The denominator degrees of freedom for statistical tests pertaining to fixed effects were calculated using the Kenward and Roger approximation [29]. Schools (nested within study sites) were treated as having random effects, while study site was treated as either a fixed or random effect depending on the analysis. Income-by-study site and level of education-by-study site interactions were used to test for differences in associations between SES and dietary patterns across study sites. Least square means for dietary patterns in each country were estimated and linear trends assessed.

Furthermore, UDP and HDP scores were dichotomized: (<0; low score and ≥0; high score) to calculate the odds of having high scores for participants in the lowest within-country income or education group compared to the highest group. Covariance parameter estimates were used to compute intraclass correlation coefficients (ICC) indicating how much of the total variance in dietary patterns was attributed to individuals, schools or study sites. Because age did not have significant main effects on dependent variables (UDP, *p* = 0.98; HDP, *p* = 0.16), it was not included in the final analytical models. Additionally, because we found no significant effects of HDI on dietary patterns, the results are not presented. *P*-values less than 0.05 were considered statistically significant.

## Results

Table 1 presents descriptive characteristics of the sample, stratified by sex and country (study site). The average age for the sample in the present study (53.7% girls) was 10.4 (0.6) years. Frequencies across the income levels and country currency ranges per within-country income level for each of the 12 countries are presented elsewhere [22]. ISCOLE study sites represented levels of HDI ranging from low (0.509, Kenya) to very high (0.929, Australia), and Gini index ranging from low (27.7, Finland) to high (63.1, South Africa).

**Table 1:** Descriptive characteristics of ISCOLE participants stratified by study site and sex (*n* = 6808)

Country (site)	n		Age (years)		HDP score		UDP score		BMI z-score	
	Boys	Girls	Boys	Girls	Boys	Girls	Boys	Girls	Boys	Girls
Australia (Adelaide)	236	272	10.4 (0.5)	10.3 (0.6)	0.18(1.0)	0.26 (0.9)	−0.26 (0.7)	−0.30 (0.7)	0.6 (1.1)	0.6 (1.1)
Canada (Ottawa)	230	321	10.0 (0.4)	10.0 (0.4)	0.44(1.0)	0.51 (1.0)	−0.40 (0.7)	−0.55 (0.5)	0.6 (1.2)	0.3 (1.2)
Finland (Helsinki, Espoo and Vantaa)	235	260	10.0 (0.5)	10.0 (0.4)	−0.18 (0.9)	−0.11 (0.9)	−0.49 (0.4)	−0.63 (0.4)	0.3 (1.1)	0.2 (1.0)
USA (Baton Rouge)	254	334	9.6 (0.7)	9.5 (0.6)	−0.06 (1.2)	−0.14 (1.1)	0.93 (1.5)	0.72(1.4)	0.9 (1.4)	0.7 (1.3)
Portugal (Porto)	294	373	10.0 (0.2)	10.0 (0.3)	0.10(1.0)	0.29(1.0)	−0.17(0.8)	−0.45 (0.5)	1.0 (1.2)	0.8 (1.1)
United Kingdom (Bath and North East Somerset)	208	257	10.4 (0.5)	10.4 (0.5)	0.02 (0.9)	−0.02 (0.9)	−0.09 (0.8)	−0.22 (0.6)	0.4 (1.1)	0.4 (1.2)
Brazil (Sau Paulo)	245	256	10.0 (0.5)	10.0 (0.5)	−0.35 (1.1)	−0.45 (1.0)	0.28 (1.1)	−0.02 (0.9)	1.0 (1.5)	0.8 (1.3)
Colombia (Bogota)	454	460	10.0 (0.6)	10.0 (0.7)	−0.48 (0.7)	−0.44 (0.7)	−0.04 (0.6)	−0.12 (0.5)	0.3 (1.1)	0.1 (1.0)
China (Tianjin)	288	254	9.4 (0.5)	9.4 (0.5)	0.04(1.0)	0.11 (0.9)	−0.11 (1.0)	−0.35 (0.8)	1.1 (1.6)	0.3 (1.3)
South Africa (Cape Town)	167	256	9.9 (0.8)	9.7 (0.7)	0.12(1.0)	0.33 (1.1)	1.33 (1.3)	1.08(1.2)	0.2 (1.2)	0.3 (1.3)
India (Bangalore)	282	320	10.0 (0.6)	10.0 (0.5)	−0.06 (1.0)	−0.07 (0.9)	0.03 (0.9)	−0.22 (0.8)	0.2 (1.5)	0.3 (1.3)
Kenya (Nairobi)	257	295	9.7 (0.7)	9.8 (0.7)	0.23(1.0)	0.30(1.0)	0.10 (1.1)	0.24(1.0)	0.1 (1.3)	0.0 (1.2)
All sites	3150	3658	10.0 (0.6)	9.9 (0.6)	−0.04 (1.0)	0.03(1.0)	0.06 (1.0)	−0.08 (1.0)	0.6 (1.3)	0.4 (1.2)

Abbreviations: *ISCOLE* International Study of Childhood Obesity, Lifestyle and the Environment, *HDP* healthy diet pattern, *UDP* unhealthy diet pattern, *BMI* body mass index

Results from the multilevel models showed that the largest proportion of total variance in dietary pattern scores occurred at the individual level, with sizable proportions at the school and site levels (individual, school, site: 62.8%; 10.8%; 26.4%) for UDP and (88.9%; 3.7%; 7.4%) for HDP, respectively. Our analyses revealed similar directionality in dietary pattern-SES gradients across the 12 sites, for both measures of SES. However, the dietary pattern-income gradients varied significantly across sites (Site-by-income interaction: p = <0.0001 (UDP), *p* = 0.05 (HDP)). Table 2 presents least square means (SE) for the relationships among dietary pattern scores and income levels stratified by country (study site). There were significant negative linear trends among UDP scores and income level in 7 of the 12 countries (i.e., an unhealthy dietary pattern was associated with lower SES). However, the relationship among HDP and income level was positive (i.e., a healthy dietary pattern was associated with higher SES) and showed significant linear trends in 5 of the 12 ISCOLE countries. Relationships among within-country dietary patterns and highest level of parental education were generally similar to those for income level (Table 3).

**Table 2:** Least square means for dietary pattern scores (UDP; HDP)^a^ by income levels for each country (site) arranged from highest to lowest HDI

Country (site)	2011 HDI (level)	Gini index (Year)	Income Level 1	Income Level 2	Income Level 3	Income Level 4	P for linear trend^b^
UDP scores by country (site)							
Australia (Adelaide)	0.929 (very high)	34.9 (2010)	−0.13 (0.10)	−0.23 (0.09)	−0.33 (0.09)	−0.46 (0.10)	0.02
Canada (Ottawa)	0.908 (very high)	32.6 (2013)	−0.23 (0.10)	−0.45 (0.09)	−0.41 (0.11)	−0.52 (0.08)	0.01
Finland (Helsinki, Espoo and Vantaa)	0.882 (very high)	27.7 (2010)	−0.46 (0.10)	−0.60 (0.10)	−0.62 (0.10)	−0.59 (0.08)	<.0001
USA (Baton Rouge)	0.837 (very high)	40.8 (2013)	1.09 (0.11)	1.27 (0.09)	0.56 (0.10)	0.51 (0.11)	0.003
Portugal (Porto)	0.819 (very high)	35.8 (2010)	−0.19 (0.09)	−0.29 (0.08)	−0.31 (0.08)	−0.46 (0.10)	0.31
United Kingdom (Bath and North East Somerset)	0.744 (very high)	36.0 (2013)	−0.14 (0.10)	−0.15 (0.09)	−0.18 (0.10)	−0.23 (0.10)	0.11
Brazil (Sau Paulo)	0.718 (high)	52.9 (2013)	0.20 (0.09)	0.11 (0.10)	0.05 (0.10)	0.01 (0.11)	<.0001
Colombia (Bogota)	0.710 (high)	55.5 (2010)	−0.06 (0.08)	−0.13 (0.08)	−0.05 (0.09)	−0.11 (0.10)	0.005
China (Tianjin)	0.687 (medium)	42.1 (2010)	−0.14 (0.14)	−0.19 (0.14)	−0.29 (0.13)	−0.23 (0.13)	0.001
South Africa (Cape Town)	0.619 (medium)	63.1 (2013)	1.47 (0.09)	0.99 (0.12)	0.98 (0.13)	0.95 (0.14)	0.74
India (Bangalore)	0.547 (medium)	33.9 (2009)	0.01 (0.12)	−0.07 (0.12)	−0.18 (0.12)	−0.26 (0.10)	0.44
Kenya (Nairobi)	0.509 (low)	47.7 (2013)	0.44 (0.10)	0.32 (0.09)	0.05 (0.09)	−0.08 (0.09)	0.22
HDP scores by country (site)							
Australia (Adelaide)	0.929 (very high)	34.9 (2010)	0.16 (0.10)	0.14 (0.09)	0.27 (0.10)	0.37 (0.10)	0.0002
Canada (Ottawa)	0.908 (very high)	32.6 (2013)	0.08 (0.10)	0.50 (0.09)	0.53 (0.12)	0.57 (0.08)	0.004
Finland (Helsinki, Espoo and Vantaa)	0.882 (very high)	27.7 (2010)	−0.13 (0.10)	−0.05 (0.11)	−0.23 (0.10)	−0.17 (0.08)	0.34
USA (Baton Rouge)	0.837 (very high)	40.8 (2013)	−0.12 (0.10)	−0.01 (0.08)	−0.11 (0.09)	−0.24 (0.11)	0.07
Portugal (Porto)	0.819 (very high)	35.8 (2010)	0.04 (0.09)	0.22 (0.08)	0.17 (0.08)	0.43 (0.09)	0.06
United Kingdom (Bath and North East Somerset)	0.744 (very high)	36.0 (2013)	−0.10 (0.10)	−0.10 (0.09)	0.16 (0.10)	0.16 (0.11)	0.27
Brazil (Sau Paulo)	0.718 (high)	52.9 (2013)	−0.46 (0.08)	−0.43 (0.10)	−0.34 (0.10)	−0.33 (0.12)	0.24
Colombia (Bogota)	0.710 (high)	55.5 (2010)	−0.51 (0.07)	−0.53 (0.08)	−0.45 (0.09)	−0.31 (0.09)	0.006
China (Tianjin)	0.687 (medium)	42.1 (2010)	−0.11 (0.12)	0.13 (0.12)	0.07 (0.11)	0.16 (0.11)	0.98
South Africa (Cape Town)	0.619 (medium)	63.1 (2013)	0.22 (0.09)	0.20 (0.11)	0.40 (0.12)	0.15 (0.16)	0.04
India (Bangalore)	0.547 (medium)	33.9 (2009)	−0.21 (0.11)	−0.10 (0.11)	−0.15 (0.12)	0.14 (0.09)	0.01
Kenya (Nairobi)	0.509 (low)	47.7 (2013)	0.23 (0.10)	0.25 (0.09)	0.12 (0.09)	0.44 (0.09)	0.45

Abbreviations: *UDP* unhealthy dietary pattern, *HDP* healthy dietary pattern

^a^Least square means for estimates of dietary patterns for each study site

^b^Test for linear trend across four income levels

**Table 3:** Least square means for dietary patterns (UDP; HDP)^a^ by education level for each country (site) arranged from highest to lowest HDI

Country (site)	2011 HDI (level)	Gini Index (Year)	Education Level 1	Education Level 2	Education Level 3	P for linear trend^b^
UDP scores by country (site)						
Australia (Adelaide)	0.929 (very high)	34.9 (2010)	−0.09 (0.13)	−0.23 (0.08)	−0.47 (0.09)	0.003
Canada (Ottawa)	0.908 (very high)	32.6 (2013)	0.29 (0.28)	−0.38 (0.09)	−0.53 (0.08)	0.0002
Finland (Helsinki, Espoo and Vantaa)	0.882 (very high)	27.7 (2010)	0.00 (0.25)	−0.61 (0.08)	−0.60 (0.09)	<.0001
USA (Baton Rouge)	0.837 (very high)	40.8 (2013)	1.43 (0.14)	0.99 (0.09)	0.69 (0.09)	0.005
Portugal (Porto)	0.819 (very high)	35.8 (2010)	−0.23 (0.08)	−0.32 (0.09)	−0.58 (0.10)	0.04
United Kingdom (Bath and North East Somerset)	0.744 (very high)	36.0 (2013)	0.10 (0.25)	−0.10 (0.08	−0.35 (0.09)	0.002
Brazil (Sau Paulo)	0.718 (high)	52.9 (2013)	0.40 (0.10)	0.05 (0.08)	0.03 (0.10)	0.07
Colombia (Bogota)	0.710 (high)	55.5 (2010)	−0.09 (0.08)	−0.11 (0.08)	−0.20 (0.11)	0.56
China (Tianjin)	0.687 (medium)	42.1 (2010)	−0.13 (0.14)	−0.22 (0.13)	−0.38 (0.14)	0.001
South Africa (Cape Town)	0.619 (medium)	63.1 (2013)	1.33 (0.09)	1.12 (0.10)	0.84 (0.14)	0.35
India (Bangalore)	0.547 (medium)	33.9 (2009)	−0.11 (0.20)	−0.17 (0.13)	−0.21 (0.11)	0.08
Kenya (Nairobi)	0.509 (low)	47.7 (2013)	0.29 (0.13)	0.18 (0.08)	0.04 (0.08)	0.01
HDP scores by country (site)						
Australia (Adelaide)	0.929 (very high)	34.9 (2010)	0.08 (0.14)	0.11 (0.08)	0.38 (0.08)	0.52
Canada (Ottawa)	0.908 (very high)	32.6 (2013)	0.31 (0.31)	0.23 (0.09)	0.51 (0.07)	<.0001
Finland (Helsinki, Espoo and Vantaa)	0.882 (very high)	27.7 (2010)	−0.13 (0.28)	−0.12 (0.07)	−0.15 (0.08)	0.93
USA (Baton Rouge)	0.837 (very high)	40.8 (2013)	−0.05 (0.15)	−0.15 (0.08)	−0.04 (0.08)	0.05
Portugal (Porto)	0.819 (very high)	35.8 (2010)	0.06 (0.07)	0.24 (0.08)	0.58 (0.10)	0.05
United Kingdom (Bath and North East Somerset)	0.744 (very high)	36.0 (2013)	−0.24 (0.28)	−0.20 (0.08)	0.27 (0.08)	0.91
Brazil (Sau Paulo)	0.718 (high)	52.9 (2013)	−0.36 (0.10)	−0.47 (0.08)	−0.35 (0.10)	0.80
Colombia (Bogota)	0.710 (high)	55.5 (2010)	−0.51 (0.07)	−0.51 (0.06)	−0.28 (0.10)	0.02
China (Tianjin)	0.687 (medium)	42.1 (2010)	−0.06 (0.11)	0.09 (0.10)	0.21 (0.12)	0.60
South Africa (Cape Town)	0.619 (medium)	63.1 (2013)	0.23 (0.09)	0.17 (0.09)	0.31 (0.14)	0.10
India (Bangalore)	0.547 (medium)	33.9 (2009)	−0.45 (0.21)	−0.19 (0.11)	0.04 (0.08)	0.07
Kenya (Nairobi)	0.509 (low)	47.7 (2013)	0.34 (0.13)	0.24 (0.07)	0.30 (0.08)	0.95

Abbreviations: *UDP* unhealthy dietary pattern, *HDP* healthy dietary pattern

^a^Least square means for estimates of dietary patterns for each study site

^b^Test for linear trend across the three levels of parental education

Our findings showed that there were no significant sex differences in within-country income gradients for either outcome (p for sex-by-income level interaction = 0.5, UDP; *p* = 0.6, HDP); thus, results were pooled for presentation. We found no significant HDI (UDP, *p* = 0.4; HDP, *p* = 0.9) nor HDI-by-income level interactions (UDP, *p* = 0.9 and HDP, *P* = 0.2) effects on dietary pattern scores (data not shown). Gini index (*p* = 0.005) but not Gini-by-income level interaction (*p* = 0.97) had significant main effects on UDP scores.

**Fig. 1:**
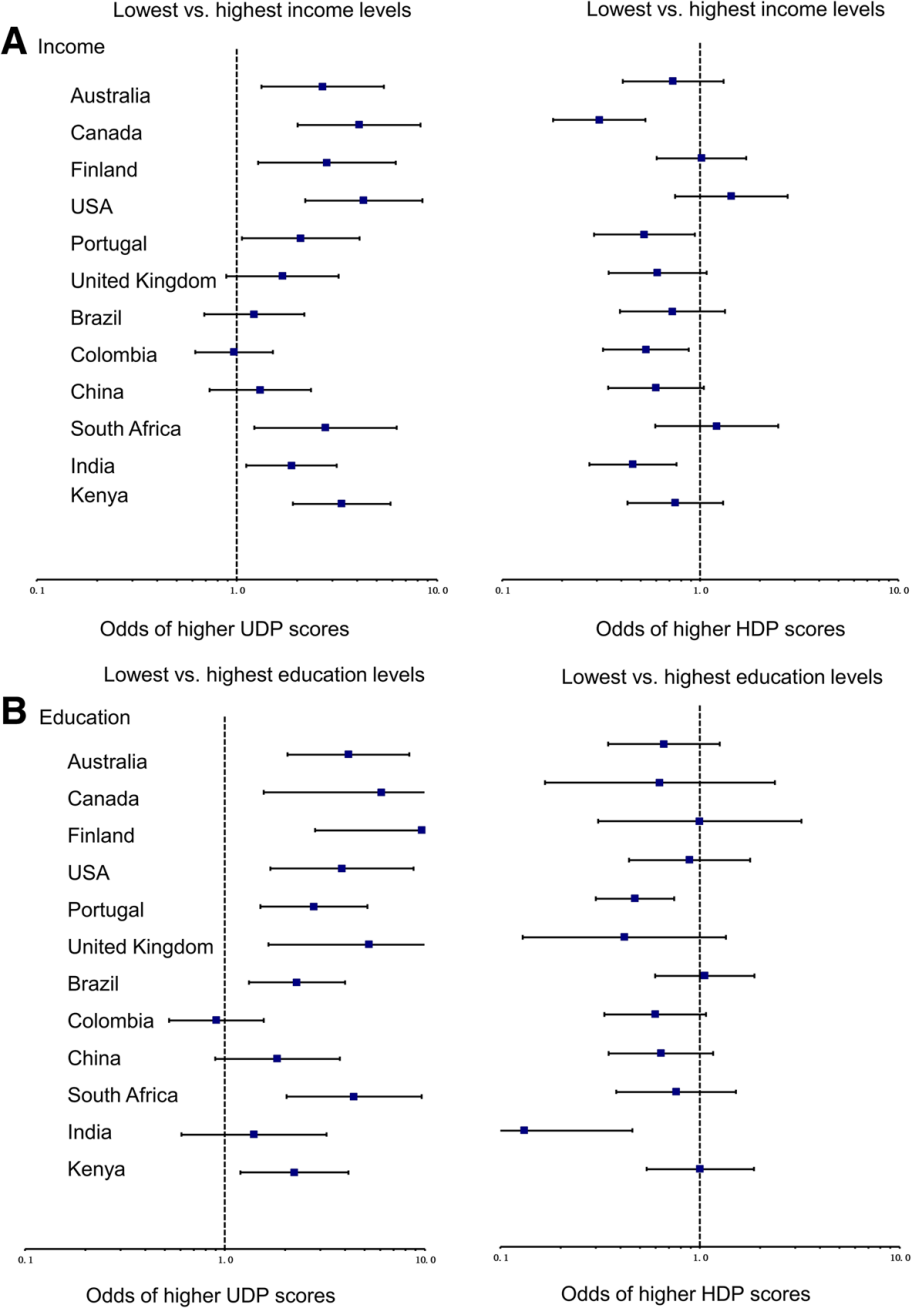
Odds of higher UDP/HDP scores for (**a**) lowest vs. highest income group and (**b**) lowest vs. highest education group in each country. Countries are arranged in descending order by HDI

Figure 1 shows the odds for participants in the lowest income (level 1) or education group (parents did not complete high school) compared to the highest group (income level 4 or parents with a degree/postgraduate degree) [17] for both higher UDP and higher HDP across the 12 ISCOLE countries. The odds of higher UDP scores for the lowest income groups were significant in Australia (2.69; 95% CI: 1.33–5.42), Canada (4.09; 95% CI: 2.02–8.27), Finland (2.82; 95% CI: 1.27–6.22), USA (4.31; 95% CI: 2.20–8.45), Portugal (2.09; 95% CI: 1.06–4.11), South Africa (2.77; 95% CI: 1.22–6.28), India (1.88; 95% CI: 1.12–3.15) and Kenya (3.35; 95% CI: 1.91–5.87). The odds for higher HDP scores were significantly lower among lowest compared to highest income groups in 4 countries: Canada (0.31; 95% CI: 0.18–0.53), Portugal (0.52; 95% CI: 0.29–0.93), Colombia (0.53; 95% CI: 0.32–0.88) and India (0.46; 95% CI: 0.27–0.76). The results were approximately similar when using either household income or highest level of parental education as proxy for SES.

## Discussion

To our knowledge, the present study was the first to examine and show within-country income-dietary pattern gradients across 12 countries from five major geographic regions of the world and widely differing human development levels. Results from the present study revealed that SES is strongly related to children’s dietary patterns across countries. Contrary to our hypothesis that within-country SES-diet patterns would show different relationships in high vs. low development countries, similar to what we have reported for obesity [17], our findings instead demonstrated that, at all levels of country-level human development, lower income or lower levels of parental education were associated with higher consumption of unhealthy foods (higher UDP scores) and lower consumption of healthy foods (lower HDP scores). Moreover, results showed significant inverse linear trends between within-country unhealthy dietary patterns and income level, and the reverse for healthy dietary patterns and income level, in at least half of the countries.

Our finding showing that, around the world, low income groups were more likely than high income groups to have higher UDP and lower HDP scores is in line with previous research findings [11, 29] and may be related to the fact that unhealthy foods generally cost less [9, 12, 30] and thus may be the only affordable choice for low income groups. Darmon and others [31] described an inverse relationship between dietary-energy density and diet cost in a sample of French adults and suggested that dry processed foods are less costly than healthier perishable meats or fresh produce. Furthermore, the positive relationship between healthy eating (HDP) and income may be explained by the fact that healthier diets have been reported to cost more [32]; hence, those in high income groups may be more likely to afford this pattern of eating. However, the potential influence of other unmeasured variables such as culture, nutrition, education, availability of healthy food choices in inner cities or low income areas, ability to afford school lunches or convenience which may be correlated to SES cannot be discounted.

The finding showing that the Gini-index was significant related to consumption of unhealthy foods (UDP) and not healthy foods (HDP) may suggest that public health efforts aimed at improving diet quality should focus on addressing unhealthy dietary patterns and promote healthy eating in conjunction with messages and strategies to reduce income inequality. This finding might partly explain why Finland, with the lowest Gini index (i.e., displaying the least income inequality) had the lowest UDP scores and South Africa with the highest Gini index (i.e., greater inequality) had the highest UDP scores. Focusing on reducing unhealthy dietary patterns may be a more urgent strategy given the documented relationship with non-communicable diseases (NCDs) [1, 3, 33]. Results from a recent multi-national study showed that consumption of healthier foods has modestly improved globally over the past two decades whereas that of unhealthier foods has significantly increased especially in low-middle-income countries (LMICs) [34].

The fact that our study showed significant within-country linear trends in dietary patterns across income levels in most of the study sites supports our first hypothesis that anticipated a similar gradient in dietary patterns across HDI levels in relation to household income. Our findings are consistent with results reported by Pechey & Monsivias’ recent cross-sectional study [29], showing greater food expenditure, which in turn was associated with healthier purchasing behaviors among adults from high income groups in the United Kingdom. The similarity in the direction of within-country diet-income gradients suggests that joint intervention strategies or public health messages aimed at improving diet quality could be similarly employed in urban and peri-urban areas across different countries. However, because our sample included only children living in urban and peri-urban areas, our results may not generalize to rural areas especially for countries at the lower end of human development where dietary patterns and access to food/agriculture may be very different to that of urban areas.

The finding that most countries in the present study display similar directionality of diet-income gradients despite being at different levels of human development contradicts our hypothesis that at lower levels of development, higher household income would be associated with unhealthy dietary patterns whereas lower household income would be associated with healthy dietary patterns. This contrary finding may indicate that in urban areas of the ISCOLE countries, the nutrition transition may be nearly complete, unlike our expectations which were partly based on apparent obesity trends in the same sample [17]. A previous study [18] found strong correlations between country-specific and pooled dietary patterns among the ISCOLE countries. Popkin and colleagues [2] described changes across LMICs in diets, moving from being traditional and converging toward a more “Western diet” characterized by highly processed, energy-dense foods that are high in fat and added sugar [35]. Reardon and colleagues [36] attributed the change in diets across LMICs to modern food distribution, which is now dominated by super-and mega-market companies that have replaced fresh or public markets throughout the developing world.

A major strength of the present study is that it had a large multinational sample of children from low-medium and high income countries at various levels of human development. Furthermore, the use of a standardized data collection protocol and rigorous quality control measures yielded high-quality data. There are important limitations to the present study worth noting including that this was a cross-sectional study and therefore causality may not be inferred. Second, the diet pattern scores were derived from self-reported data, which has potential for recall bias, and the instrument we used did not account for volume of ‘healthy’ or ‘unhealthy’ food consumption. Furthermore, food consumption was assessed with an exclusive list of food items (quite Western orientated) and thus may not have captured all aspects of diets around the world.

However, the use of a valid and reliable instrument [37] was intended to minimize error and bias. Third, the ISCOLE sample was drawn from urban and peri-urban areas, thus our results may not be generalizable to rural areas, especially in LIMCs where dietary patterns of rural vs. urban children are more likely to be significantly different [16]. Finally, the potential confounding effects of unmeasured variables cannot be discounted.

## Conclusion

Our study provides evidence showing that children’s dietary patterns are related to their SES. Our findings demonstrate that, around the world, unhealthy dietary patterns are strongly related to SES with those in lower income groups having higher UDP scores hence the need to link policies or intervention strategies aimed at improving diet quality to the economics of food. Given the link between unhealthy diet and NCDs, there is a need to better understand these relationships. Future studies should explain and breakdown these relationships further. It is also important for future research to replicate and extend this work to include rural areas where dietary patterns may be very different from those of urban and peri-urban areas. Furthermore, intervention studies may be needed to assess causation.

## Additional files

Additional file 1: Appendix S2. ISCOLE Diet and Lifestyle questionnaire (Adapted from the Health Behavior in School-aged Children Survey). (DOCX 248 kb)

Additional file 2: Appendix S3. Ethics Boards Approvals for each of the 12 ISCOLE sites. (DOC 23 kb)

## Abbreviations

BMI: Body mass index
FFQ: Food frequency questionnaire
HDI: Human development index
HDP: Healthy diet pattern
ISCOLE: International study of childhood obesity, lifestyle and the environment
LMICs: Low-middle-income countries
NCDs: Non-communicable diseases
SES: Socio-economic status
UDP: Unhealthy diet pattern
