# Supplementary material for: Socioeconomic status and dietary patterns in children from around the world: different associations by levels of country human development?
Source: BMC Public Health. 2017 May 16;17:457. doi: 10.1186/s12889-017-4383-8 (PMC5434585; doi:10.1186/s12889-017-4383-8)
Supplement: Supplementary file 2 — Appendix S3. Ethics Boards Approvals for each of the 12 ISCOLE sites. (DOC 23 kb) [file 12889_2017_4383_MOESM2_ESM.doc]

Appendix S3: List of Research Ethics Boards for each study site

1. University of South Australia Human Research Ethics Committee (Australia)
2. Research Ethics Committee of the Municipal Health of Sao Caetano do Sul—Prima (Brazil)
3. Children’s Hospital of Eastern Ontario Research Ethics Board (Canada)
4. Biomedical Ethics Committee of Tianjin Women’s and Children’s Health (China)
5. Universidad de los Andes Committee on Research Ethics (Colombia)
6. Ethics Committee of the Hospital District of Helsinki and Uusimaa (Finland)
7. St. John’s Medical College and Hospital Institutional Ethical Review Board (India)
8. Kenyatta University Ethics Review Committee (Kenya)
9. Ethics Committee University of Porto (Portugal)
10. University of Cape Town Health Sciences Faculty Human Research Ethics Committee (South Africa)
11. University of Bath Research Ethics Committee for Health (UK)
12. Pennington Biomedical Research Center Institutional Review Board for Research with Human Subjects (USA)
